# Single-staged uniportal VATS in the supine position for simultaneous bilateral primary spontaneous pneumothorax

**DOI:** 10.1186/s13019-017-0591-7

**Published:** 2017-05-15

**Authors:** Kyung Soo Kim

**Affiliations:** Department of Thoracic and Cardiovascular Surgery, Seoul St. Mary’s Hospital, College of Medicine, The Catholic University of Korea, Banpo-daero 222, Seocho-gu, Seoul, 06591 Republic of Korea

**Keywords:** Uniportal, Single port, Pneumothorax, Thoracoscopic surgery, Video Assisted Thoracic Surgery (VATS), Supine position

## Abstract

**Background:**

Simultaneous bilateral primary spontaneous pneumothorax (SBPSP) is rare, but requires surgery on both sides, in patients with definite bilateral bullae to prevent life-threatening conditions. Recently, uniportal video-assisted thoracoscopic surgery (VATS) has been widely accepted as a less invasive technique for the treatment of pneumothorax. Thus, we introduced single-staged uniportal VATS technique in the supine position, for the management of two cases of SBPSP.

**Case presentation:**

A 17-year-old boy presented with bilateral spontaneous pneumothorax and he underwent single-staged uniportal VATS in the supine position. Single wide draping in consecutive bilateral approaches removes the needs of changing patients’ position. Whole thoracoscopic procedure for wedge resection of bullae lesions was conducted without difficulty. The total operation time took 65 min and the patient discharged 3 days after the operation. The patient was followed for 24 months without recurrence of both sides. Another 18-year-old boy was admitted with bilateral spontaneous pneumothorax and single-staged uniportal VATS was also performed in the supine position. The total operation time took 79 min and the patient discharged on postoperative day 4. He was followed for 19 months without recurrence of both sides.

**Conclusions:**

Single-staged uniportal VATS approach yielded satisfactory results from simplicity that not requires position change compared to conventional multi-ports VATS in the lateral position, and with better cosmetics. This technique is thought to be a feasible procedure in selective patients with SBPSP or with contralateral bullae for preventive role.

**Electronic supplementary material:**

The online version of this article (doi:10.1186/s13019-017-0591-7) contains supplementary material, which is available to authorized users.

## Background

Bilateral primary spontaneous pneumothorax, either non-simultaneous or simultaneous, has a reported incidence of 7.8 to 20% among pneumothorax patients [1]. Simultaneous bilateral primary spontaneous pneumothorax (SBPSP) is a rare condition with an incidence of 1%, but single-staged surgery is required on both sides of patients with definite bilateral bullae to prevent the life-threatening condition [2]. Recently, single incisional video-assisted thoracoscopic surgery (VATS) has been accepted for the treatment of primary spontaneous pneumothorax [3–5]. Treatment of bilateral pulmonary lesions has been reported to be effective using single-staged approach in the lateral position on position change [6]. Bilateral single-port sympathicotomy has also been introduced as a feasible technique in the supine position [7]. Thus, we introduced a single-staged uniportal VATS technique in the supine position for treatment of two cases of SBPSP, and evaluated technical feasibility with surgical outcomes.

## Case presentation

### Case 1

**Fig. 1:**
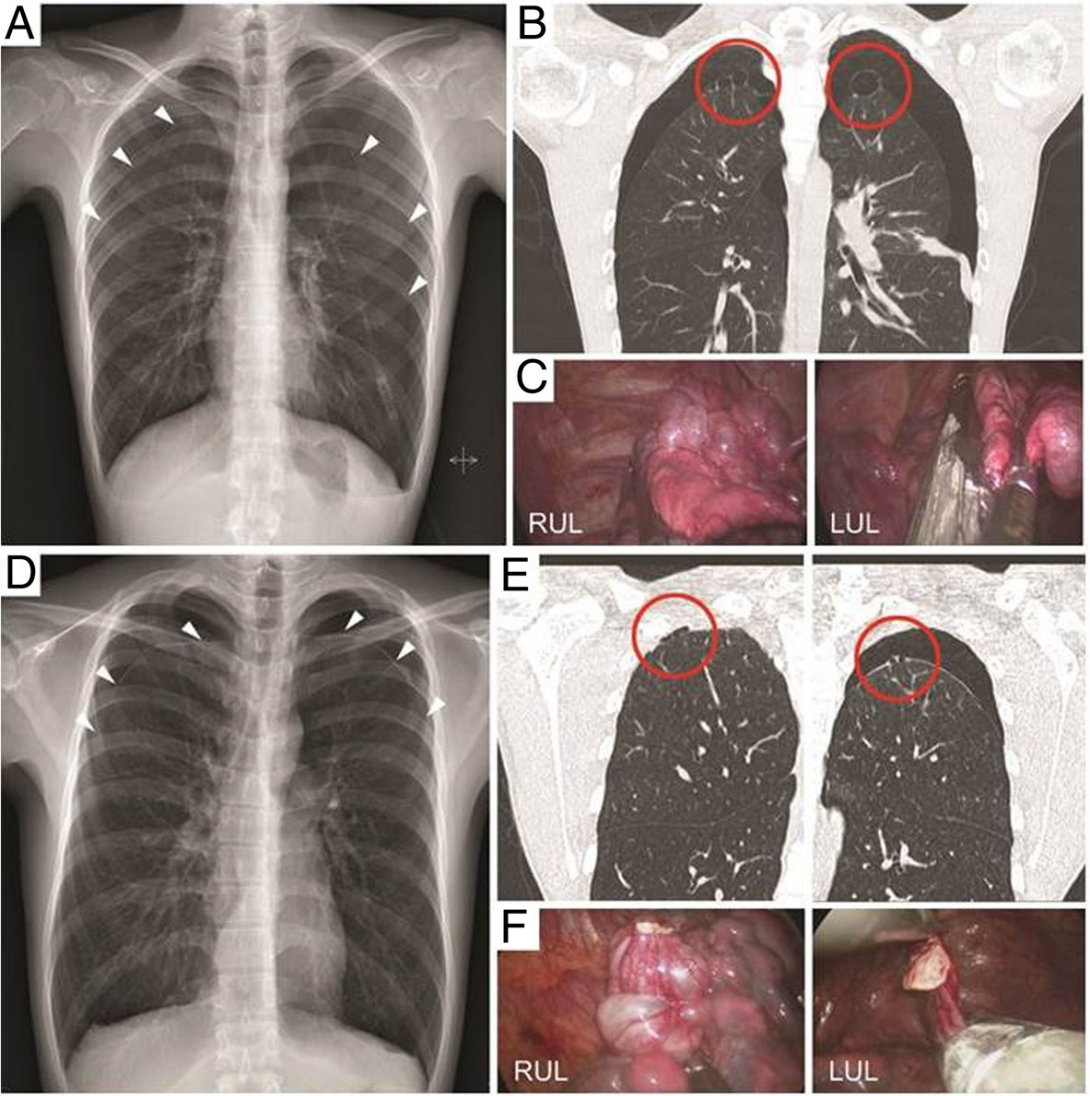
**a**, **d** Chest x-ray shows bilateral spontaneous pneumothorax of case 1 and 2 (*white arrow head*). **b**, **e** Chest tomography image shows multiple, variable-sized apical bullae on both lung apices (*red circle*). **c**, **f** Wedge resection of bilateral bullae lesions using endostaplers under 5-mm, 30° thoracoscopic view

**Fig. 2:**
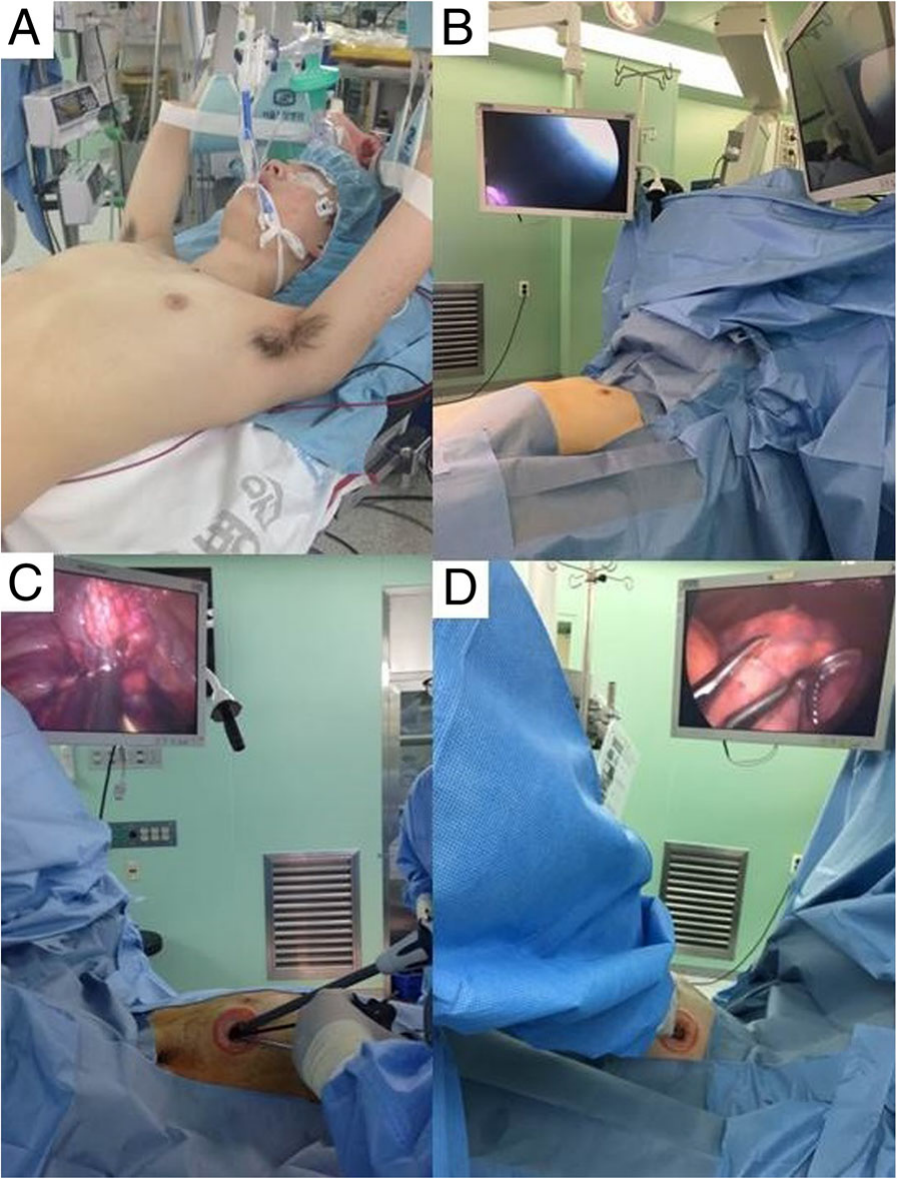
**a** Patient positioning for bilateral, staged-operation. In a supine position, both arms were hung to the overhead strut of the ether screen using arm slings, and a cushion was packed below the back for chest elevation. **b** Discrete high definition monitors were set up bilaterally for sequential operation without changing of monitor position. **c** Surgical image of instrumentation during right-side thoracoscopic wedge resection. **d** The simultaneous thoracoscopic wedge resection was continued on the contralateral side (left side) and thoracoscopic view shows saline merging test in the Trendelenburg position

A 17-year-old boy presented with chest discomfort for 1 week. A chest X-ray and preoperative high-resolution computed tomography (HRCT) revealed images of SBPSP with multiple apical bullae on both lung apices (Fig. 1a-b). Under general anesthesia with double-lumen endotracheal intubation for sequential, selective lung ventilation, the patient was positioned supine with arms hanging to the overhead strut of the ether screen using the arm slings. Then, cushion pads were packed below the midline of the back for chest elevation to avoid collision of thoracoscopic instruments and the operating table (Fig. 2a). After transverse single draping to expose the bilateral chest wall simultaneously, the operative side was slightly elevated by table tilting to the opposite side. Two discrete high definition monitors were set up for sequential operation without changing of monitor position (Fig. 2b). Then, a minimal skin incision (less than 2.5 cm in length) was made in the fourth intercostal space at the anterior axillary line. First, full thoracoscopic inspection was conducted from the apex to the base using a 5-mm, 30° thoracoscope and thoracoscopic assistant stands on the operator’s sides of the same view-point. Exploration of entire lung was possible by lung retractions using endo-grasper under rotated thoracoscopic view (Fig. 2c). An identified apical bullae lesion of the upper lobe was held with grasping forceps and resected with endostaplers (Echelon 60 Endopath stapler; Ethicon Endosurgery Corp., Cincinnati, OH, USA) through the uniport (Fig. 1c). Pleural abrasion was not performed, and a saline merging test by temporary lung expansion after infusion of warm physiological saline in the Trendelenburg position confirmed no air leakage. An absorbable polyglycolic acid sheet (Neoveil; Gunze, Kyoto, Japan) was covered with fibrin glue (Beriplast-P combiset; CSL Behring GmbH, Marbug, Germany). Then, a 16 Fr. chest tube was placed at the anterior part of the uniport. After confirmation of no wound bleeding into direct thoracoscopic view, intercostal nerve block was conducted. Then, the wound was closed layer by layer using an absorbable suture in a running fashion with 2-0 and 3-0 Braided synthetic absorbable suture (Polysorb^TM^, Covidien, Mansfield, MA) under both lung ventilation, and the skin was approximated with 4-0 barbed absorbable suture material (V-Loc™ Absorbable Wound Closure Device product line, Covidien, Mansfield, MA). The same procedures were conducted on the contralateral side via a 2.5 cm single incision in the fourth intercostal space at the anterior axillary line (Fig. 2d). Under the selective one lung ventilation by experienced anesthesiologists, the table was slightly tilted to the opposite direction without changing position into lateral decubitus. During the single-staged operation on the contralateral side, function of the chest tube on opposite side is well detectable, confirming neither air leakage nor tube compression of the chest wall. The total operation time took 65 min without any intra-operative events. Intravenous patient controlled analgesia was applied for relief from postoperative pain. Bilateral chest tubes were removed on postoperative days 1 and 2, serially. The patient recovered uneventfully and discharged 3 days after the operation. The patient was followed at the out-patient clinic for 24 months without a recurrence of pneumothorax on both sides.

### Case 2

An 18-year-old boy was conservatively managed for a bilateral minimal amount of pneumothorax during following up of 6 months at the out-patient clinic. He visited the hospital again complaining of chest discomfort. A chest X-ray revealed bilateral spontaneous pneumothorax, and an HRCT revealed multiple, variable-sized apical bullae on both lung apices (Fig. 1d-e). An operation was performed to control recurrent pneumothorax with identified bilateral definite bullae. The operative procedures were similar to the Case 1 except division procedure of the adhesion band on the apex of the right upper lobe (Fig. 1f, Additional file 1: Video S1). The total operation time was 79 min without any intra-operative events. Bilateral chest tubes were removed on postoperative days 1 and 3. The patient discharged on postoperative day 4 without complications. He was followed for 19 months without recurrence of both sides.



**Additional file 1: Video S1.** Operative procedures of single staged uniportal VATS technique for SBPSP. (MP4 11779 kb)


## Discussion

Simultaneous bilateral primary spontaneous pneumothorax (SBPSP) is a rare clinical event, with a reported incidence as low as 1% [2]. SBPSP may develop in an emergency situation, which requires either urgent chest tube insertion or surgery in cases with definite bilateral bullae to prevent life-threatening conditions. SBPSP has been treated using trans-sternal, bilateral thoracotomy, mini-thoracotomy, or sequential VATS approach [8, 9]. Several reports discussed on a bilateral VATS approach to spontaneous pneumothorax in the lateral position, also multi-ports VATS for SBPSP in the supine position demonstrated its efficacy and low morbidity [10, 11]. Recently, uniportal VATS have gained feasibility using minimal skin incision as well as its lower morbidities due to the least involvement in intercostal space compared to multi-ports VATS [12, 13]. Thus, we transferred the less invasive VATS approach in the supine position into the treatment of SBPSP.

Several advantages were demonstrated by our technique comparing to conventional multi-ports VATS in lateral decubitus position. In supine position, single wide draping in consecutive bilateral approaches after slight tilting of the operation table in the opposite direction removes the needs of changing patients’ position. Thus, the whole procedure can be performed simpler without time-consuming re-draping, position change or possible chest tube compression, which may be required in a lateral position. The positioning of a patient is simple in that both arms are hung overhead, and a cushion is packed below the back to avoid collision of thoracoscopic instruments with the operating table. In addition, this procedure also helps to widen the narrow intercostal spaces [14]. An alternating inflation technique using pneumatic cuffs was introduced and we thought this may be useful in our technique for treatment of SBPSP. However, excessive chest wall elevation was not required because the cushion pad of the back was enough for full exposure to the surgical field without disturbance of instrumental manipulation [15].

Bertolaccini et al. demonstrate the geometric and ergonomic advantages of uniportal VATS approach compared to the standard three-port approach in a lateral position which resulting in greater fatigue [16]. In supine position, thoracoscopic surgical instruments including endostaplers can also be introduced into parallel approaches from the same level of uniport. Thus, articulation technique using endostapler is less needed in supine position compared to the conventional VATS in a lateral decubitus position that surgeon may feel more comfortable to manipulate the instruments as we experienced. Total operation time may also be shortened by saving time for reconfirming the double-lumen tube position after position change and time for multiple wound closure. The saline submerging test can also be performed without technical difficulties by using Trendelenburg position with 15–30° using less amount of saline infusion.

The trans-mediastinal thoracoscopic approach using multi-ports already has been demonstrated for simultaneous resection for bilateral bullae lesions with satisfactory results [17–19]. In a single port surgery, it is thought to be difficult to manipulate the thoracoscopic instruments for mediastinal dissection through the minimal single incision.. However, the single-stage, uniportal trans-mediastinal approach might also be challengeable because it may be more easily accessible to the supine position due to the ergonomic advantages, as mentioned above.

In our cases, chest tubes were not inserted prior to the operations because both patients had no underlying pulmonary disease and showed stable vital signs without dyspnea. We considered the possible situations of desaturation during selective one lung ventilation. We thought that air tapping or chest tube insertion on the opposite side can be more easily accessible during a one-side VATS by wide draping in the supine position, compared to the conventional VATS that require position change in the  lateral position. We also considered that more collapsed lung is acceptable to the first operation but it is surgeons’ preference according to the patient’s condition. If the patient had the chest tube on any side with lung expansion, contralateral lung should be the first choice of approach. In our cases, we could perform the whole sequential procedure without placement of additional ports or conversion to thoracotomy because patients were tolerable without desaturation under multi-monitoring by experienced anesthesiologists.

The possibility of using VATS for preventive operations on the contralateral lung lesions for pneumothorax is still controversial. However, more than 50% of ipsilateral pneumothorax patients show contralateral blebs or bullae on CT scan [20]. Furthermore, several reports have suggested its cost-effectiveness for saving subsequent hospitalization or operation, and beneficial effect on preventing contralateral occurrence (0–7%) due to its high recurrence rates (18–50%) [21, 22]. Thus, our technique is thought to be a useful option even for the management of ipsilateral pneumothorax cases of definite bilateral bullae on CT scans.

## Conclusions

Single-staged, uniportal VATS technique in the supine position is thought to be a feasible procedure in selective patients with SBPSP or with contralateral bullae for preventive role.

## Abbreviations

HRCT: High-resolution computed tomography
SBPSP: Simultaneous bilateral primary spontaneous pneumothorax
VATS: Video-assisted thoracoscopic surgery
